# Cortical and thalamic electrode implant followed by temporary continuous subthreshold stimulation yields long-term seizure freedom: A case report

**DOI:** 10.1016/j.ebr.2020.100390

**Published:** 2020-09-02

**Authors:** Juan Luis Alcala-Zermeno, Nicholas M. Gregg, Jamie J. Van Gompel, Matt Stead, Gregory A. Worrell, Brian Nils Lundstrom

**Affiliations:** a Department of Neurology, Mayo Clinic, Rochester, MN, USA; b Department of Neurologic Surgery, Mayo Clinic, Rochester, MN, USA; c Dark Horse Neuro, Inc., Bozeman, MT, USA

**Keywords:** Drug-resistant epilepsy, Seizure freedom, Chronic subthreshold cortical stimulation, Chronic subthreshold stimulation, Neuromodulation, Anterior thalamic nucleus

## Abstract

Neuromodulation strategies that target the epileptogenic network are options for treating focal drug-resistant epilepsy. These brain stimulation approaches include responsive neurostimulation and more recently, chronic subthreshold stimulation. Long-term seizure freedom with neuromodulation is uncommon. Seizure control typically requires ongoing froms of electrical stimulation. Here, we present the case of a patient implanted with three cortical electrodes targeting the inferior frontal lobe, insula, and one subcortical electrode targeting the ipsilateral anterior thalamic nucleus. This patient received continuous subthreshold electrical stimulation to the frontal electrodes for 7 months, at which time stimulation was inadvertently stopped. He has now been free of seizures for 42 months. This case suggests the possibility that neuromodulation can alter epileptogenic networks and lead to seizure freedom without ongoing electrical stimulation.

## Highlights

• Brain stimulation can treat drug-resistant focal seizures but rarely leads to seizure freedom.
• Ongoing brain stimulation is typically thought necessary to provide therapeutic effect.
• We present a patient that achieved seizure freedom after several months of brain stimulation.
• The patient has maintained seizure freedom for over three years following inadvertent cessation of brain stimulation.
• This case suggests the possibility that brain stimulation can permanently modulate neural circuitry.

## 1 Introduction

Focal epilepsy is the most common type of epilepsy encountered in population-based studies [1]. Approximately 30% of epilepsy patients do not achieve seizure freedom with antiseizure drugs (ASD) [2]. With focal drug-resistant epilepsy (DRE), resection of the epileptogenic focus offers the best chance for seizure freedom. However, when seizures arise from eloquent cortex, a surgically inaccessible zone, or a widespread area, resective surgery may not be feasible. Neuromodulation with brain stimulation offers alternatives including deep brain stimulation (DBS) of the anterior nucleus of the thalamus and responsive neurostimulation (RNS) althrough seizure freedom is rare with neuromodulation.

Chronic subthreshold stimulation (CSS) is an investigational approach that includes continuous stimulation of the cortex [3]. Typically, CSS includes a period of trial stimulation with temporary electrodes to optimize the stimulation location and parameters prior to permanent electrode implant [4]. Four electrodes with a total of 16 contacts are often implanted [5]. We present the case of a patient with focal DRE who was implanted with three electrodes targeting the right inferior frontal gyrus, frontal operculum and insula and one electrode targeting the anterior nucleus of the ipsilateral thalamus. Reporting was approved by the Mayo Clinic Institutional Review Board.

## 2 Case

A 26-year-old right-handed man presented with drug-resistant focal epilepsy that started at age 16. His history was remarkable for a right hemispheric perinatal stroke complicated by neonatal seizures and hemiparesis that resolved during childhood. The seizure semiology consisted of a somatosensory aura described as “begin dunked in ice cold water” followed by occasional non-versive right head turn, oral automatisms, and grunting and flailing movements of both arms and legs with impaired awareness and rapid postictal return to baseline. Seizures lasted about 20 s. Their frequency was about six events per month with clusters that could reach up to 60 seizures in a few hours every 4–6 weeks. At presentation, he was on levetiracetam 1500 mg in the morning and 2000 mg at bedtime as well as lamotrigine 300 mg twice a day. He had previously tried zonisamide and lacosamide without benefit.

Interictal scalp EEG did not show any epileptiform discharges, and ictal EEG was non-lateralizing due to abundant muscle artifact. His hypermotor semiology suggested an extra-temporal etiology. MRI showed chronic findings of a right middle cerebral artery infarction with associated encephalomalacia and gliosis. Subtraction Ictal SPECT Co-registered to MRI (SISCOM) showed hyperperfusion in the right hemisphere near the area of encephalomalacia. Magnetoencephalography (MEG) showed right hemispheric dipoles clustered over the right frontal operculum and posterior frontal corona radiata. Neuropsychologic evaluation revealed visual memory difficulties, constructional apraxia, and executive dysfunction with normal verbal memory and language.

**Fig. 1:**
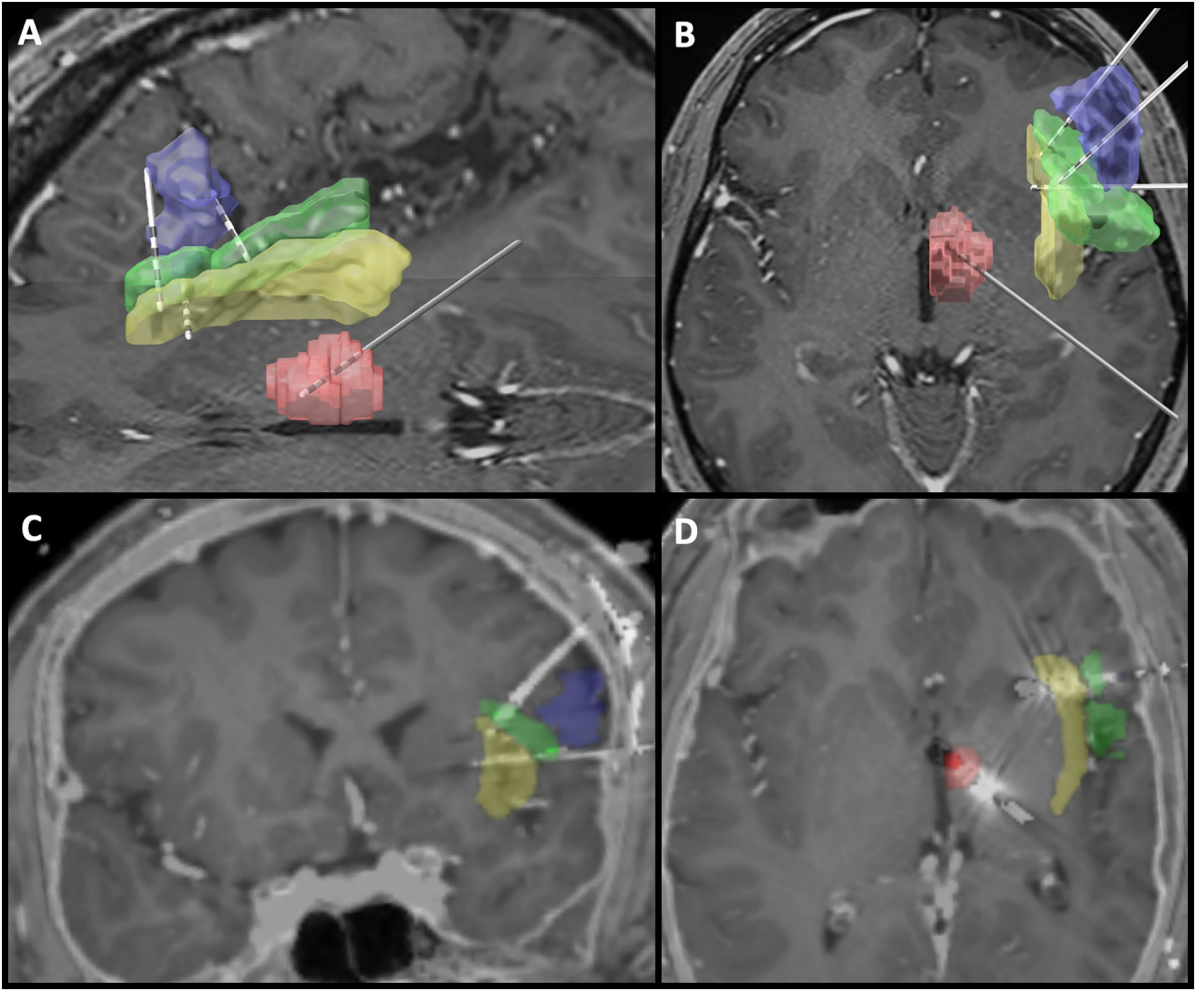
Visualization of implanted electrodes and stimulation targets. A) Sagittal and B) axial views with 3D rendering of stimulated targets. C) Coronal and D) axial plane images of the coregistered implant, with color coded stimulation targets. Red: anterior thalamus; yellow: insula; green: frontal and central operculum; blue: inferior frontal gyrus pars opercularis. Images are show in neurological orientation (right sided structures located to the right). Images were generated using Lead-DBS software [6] and thalamic [7] and cortical [8] deformable atlases.

**Fig. 2:**
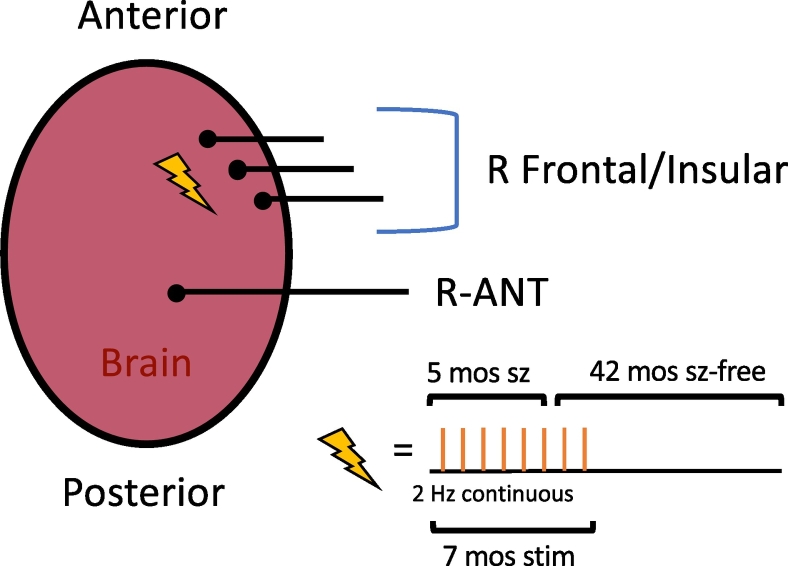
Four electrodes were permanently implanted for chronic subthreshold stimulation. Three leads targeted the right frontal head region, and one lead targeted the right anterior nucleus of the thalamus. Continuous stimulation was started in the right frontal leads at 2 Hz. After five months of stimulation the patient reported seizure freedom. Two months later stimulation of all leads ceased.

The patient underwent stereoelectroencephalography (sEEG) using 13 depth electrodes probing the right hemisphere including the temporal mesial structures as well as the right frontal head region surrounding the area of encephalomalacia. The most frequent interictal epileptiform discharges (IEDs) were present in three electrodes surrounding the area of encephalomalacia including the right inferior frontal gyrus and insula. Approximately 40 stereotypical seizures were observed without clear associated seizure activity. However, many seizures were preceded by a brief burst of spikes coming from the electrode targeting the right anterior inferior frontal gyrus near the area of encephalomalacia and insula. It was felt that this electrode may be near the seizure onset zone. sEEG electrodes were removed. Resection was not pursued as it was not felt that the seizure onset zone had been clearly localized. After discussions in a multidisciplinary conference and with the patient, it was decided to implant permanent electrodes surrounding this area, and externalize the leads in order to proceed with trial stimulation for CSS. Six months later three Medtronic 3387 leads (Medtronic, Minneapolis, MN, USA) were implanted in the right perisylvian area, an orthogonal lead in the inferior frontal gyrus and two superior-to-inferior leads targeting the anterior and posterior insula. A fourth 3387 lead was implanted in the right anterior nucleus of the thalamus (ANT) (Fig. 1). A postoperative CT scan was unremarkable for surgical complications. A trial of therapeutic continuous extra-operative electrical stimulation was initiated by connecting these leads to an external neurostimulator (Medtronic 37022). Over the course of approximately 24 h, IEDs were largely reduced using stimulation parameters of 2.5 V amplitude, 2 Hz frequency, and 120 μs pulse width applied to three electrodes surrounding the right frontal operculum. Leads were then internalized and connected to a Medtronic Restore internal pulse generator. Prior to hospital discharge, chronic stimulation for each of the three right frontal electrodes was started at 2 V amplitude, 2 Hz frequency and 120 μs pulse width in a bipolar configuration (a single distal anode and two or three proximal neighboring cathodes for each electrode). No stimulation was started for the right ANT lead (Fig. 2). The patient remained seizure-free for 48 h prior to hospital discharge.

Soon after initiation of stimulation seizure frequency decreased by more than 50%. During the subsequent four months, stimulation amplitude was increased to 2.2 V. Five months after implantation, the patient and his wife reported seizure freedom. Routine clinical follow-up one year later included a routine EEG during wakefulness and sleep performed that was normal with no evidence of IEDs or background slowing. The stimulation device was not interrogated during this visit as the patient remained seizure-free. His next follow-up visit occurred three years following implantation. At that time, it was realized that his stimulation device had been off since approximately seven months after implantation, or approximately two months after the patient's seizures stopped, and had remained off. Device interrogation and assessment showed no signs of malfunction or compromise of lead integrity. In discussion with the patient, there was no history of significant trauma. The patient may have inadvertently turned the device off with his patient programmer. Given his continued seizure freedom, stimulation was not restarted. At his last follow-up, three and a half years after implantation, the patient remained seizure-free. Throughout this time period, the patient did not report any new neurological symptoms. His anti-seizure medications remained unchanged, and he reports no medication side effects. In summary, the patient reports ongoing seizure freedom that appears to be the result of persistent neuromodulation caused by temporary brain stimulation.

## 3 Discussion

We describe a patient who received continuous subthreshold cortical stimulation, continued to have seizures, and then became seizure-free while receiving stimulation. He remained seizure-free despite unintentional deactivation of his device and has remained seizure-free since that time approximately three years ago.

Clinical benefit from electrode implantation alone is well-recognized in DBS for movement disorders with associated functional neuroimaging evidence of neurophysiologic changes [9]. Similarly with regard to epilepsy, patients implanted with either DBS or RNS can have 20–30% seizure frequency reduction from electrode implantation [10,11]. With RNS, this implant effect correlates with observed neurophysiological changes during the first five months [12], and is considered a temporary phenomenon.

In rare cases, the beneficial effect of electrode implantation is long-lasting. A small controlled trial of 13 epilepsy patients undergoing centromedian thalamic stimulation showed that two patients became seizure-free after electrode implantation; one remained seizure-free (5 years) and the other had recurrence at 13 months [13]. Prolonged seizure freedom prior to stimulation initiation has also been observed following implantation of RNS hardware [14]. In addition, seizure freedom following invasive monitoring with sEEG has been reported in about 0.5% of cases [15,16]. Often there is evidence of damage along the depth electrode tracts which could indicate unintentional lesioning of the epileptogenic network and neighboring fibers responsible for seizure spread [17,18]. This can also be observed without imaging evidence of a parenchymal lesion [19] as microscopic changes in neural tissue after electrode insertion could disrupt epileptogenic neurons and fibers [20].

Although electrode implantation may have contributed to seizure freedom, electrical stimulation may also have a neuromodulatory effect such that ongoing electrical stimulation was no longer required. Available evidence from DBS-ANT and RNS pivotal trials indicate that after the implant effect passes, the beneficial effect from neurostimulation increases over time [21,22]. Despite this, seizure freedom remains rare. In the SANTE trial, 16% of individuals reported a seizure-free period of at least six months and one patient was seizure-free for more than four years in the extended follow-up period [10,22]. For RNS, 23% of participants were seizure-free for at least six months and 13% for one year [11,21]. For CSS, periods of freedom from disabling seizures with ongoing stimulation were reported in 50% of patients for six months and 40% for one year, although these data come from limited patient numbers in a smaller retrospective cohort [3].

The particular neurostimulation paradigm of this patient is somewhat unique given the presence of three depth electrodes targeting neocortex surrounding the seizure focus with continuous stimulation. These electrodes delivered approximately 37 million stimulation pulses over seven months prior to stimulation cessation. The reason for his continued seizure freedom three and a half years later remains unclear. However, this case report supports the possibility that neuromodulation can exert long-lasting changes to neural networks.

## Ethics statement

This research was conducted under approval of the Mayo Clinic Institutional Review Board.

## Funding

Research was support by NIH NINDS K23NS112339 (BNL), American Epilepsy Society Research & Training Fellowship for Clinicians (NMG), and 10.13039/100000002NIH 10.13039/100000065NINDS R01NS92882 (GAW).

## Declaration of competing interest

JJVG, MS, GAW, and BNL are named inventors for intellectual property licensed to Cadence Neuroscience Inc., which is co-owned by Mayo Clinic. BNL has waived contractual rights to royalties. JJVG, GAW, and BNL are investigators for the Medtronic Deep Brain Stimulation Therapy for Epilepsy Post-Approval Study (EPAS). JJVG, GAW and BNL are investigators for Mayo Clinic Medtronic NIH Public Private Partnership (UH3-NS95495). JJVG, MS and GAW assisted in a Mayo Clinic Medtronic sponsored FDA-IDE for the investigational Medtronic Activa PC + S device. Mayo Clinic has received consulting fees on behalf of BNL from Medtronic and Philips.
